# Keap1 inhibition sensitizes head and neck squamous cell carcinoma cells to ionizing radiation via impaired non-homologous end joining and induced autophagy

**DOI:** 10.1038/s41419-020-03100-w

**Published:** 2020-10-21

**Authors:** Sara Sofia Deville, Susanne Luft, Maria Kaufmann, Nils Cordes

**Affiliations:** 1 OncoRay—National Center for Radiation Research in Oncology, Faculty of Medicine and University Hospital Carl Gustav Carus, Technische Universität Dresden, Dresden, Germany; 2 Helmholtz-Zentrum Dresden—Rossendorf, Institute of Radiooncology—OncoRay, Dresden, Germany; 3 Department of Radiotherapy and Radiation Oncology, Faculty of Medicine and University Hospital Carl Gustav Carus, Technische Universität Dresden, Dresden, Germany; 4 German Cancer Consortium (DKTK), Partner Site Dresden, Dresden, Germany; 5 German Cancer Research Center (DKFZ), Heidelberg, Germany

**Keywords:** Head and neck cancer, Cell death

## Abstract

The function of Keap1 (Kelch-like ECH-associated protein 1), a sensor of oxidative and electrophilic stress, in the radiosensitivity of cancer cells remains elusive. Here, we investigated the effects of pharmacological inhibition of Keap1 with ML344 on radiosensitivity, DNA double-strand break (DSB) repair and autophagy in head and neck squamous cell carcinoma (HNSCC) cell lines. Our data demonstrate that Keap1 inhibition enhances HNSCC cell radiosensitivity. Despite elevated, Nrf2-dependent activity of non-homologous end joining (NHEJ)-related DNA repair, Keap1 inhibition seems to impair DSB repair through delayed phosphorylation of DNA-PKcs. Moreover, Keap1 inhibition elicited autophagy and increased p62 levels when combined with X-ray irradiation. Our findings suggest HNSCC cell radiosensitivity, NHEJ-mediated DSB repair, and autophagy to be co-regulated by Keap1.

## Introduction

Survival rates of patients with head and neck squamous cell carcinomas (HNSCC) remain to be optimized^1–3^. In addition to conventional radiochemotherapy, great efforts were undertaken to identify both biomarkers and potential therapeutic target molecules. In a high-throughput screen in three-dimensionally grown HNSCC cell lines, we recently identified Keap1 (Kelch-like ECH-associated protein (1) as critical regulator of cellular radiosensitivity^4^. The Keap1/Nrf2 (Nuclear factor (erythroid-derived-2)-like (2) signaling axis senses free radicals and protects the cell during excessive oxidative and electrophilic conditions^5^. Under non-stressed conditions, Keap1 determines Nrf2 activity by binding and polyubiquitination, followed by proteosomal degradation. During cellular stress like exposure to X-ray irradiation, Nrf2 is released and accumulates in the nucleus where it functions as transcription factor for cytoprotective antioxidant genes^5^. A prevalence and prognostic value of Keap1 and Nrf2 mutations are well known in cancer including HNSCC^6–8^.

Mechanistically, the Keap1/Nrf2 axis has been reported to be involved in various cell functions such as DNA repair or autophagy^9^. In DNA repair, the production of various kinds of radicals is closely associated with DNA damage and Keap1 takes part in the maintenance of the cell’s homeostatic state. In general, the most lethal damages generated by ionizing radiation (IR) are DNA double-strand breaks (DSBs)^10^. Cells comprise two major cellular machineries to repair these DNA lesions, i.e., non-homologous end joining (NHEJ) and homologous recombination (HR)^11,12^. While NHEJ is an error-prone process active throughout the entire cell cycle, HR is mostly regarded as error-free repair process confined to the S/G2 cell cycle phases. After DSB recognition by the DNA damage response (DDR) proteins Mre11, Rad50, and Nbs1 (MRN complex), ATM is activated and subsequently phosphorylates H2A histone family member X (H2AX). During NHEJ, Ku70/Ku80 heterodimers are recruited to broken DNA ends followed by the activation of DNA protein kinase catalytic subunit (DNA-PKcs)^10^. Owing to its central process for cell survival, targeting the DNA repair machinery is still considered as powerful approach in cancer treatment obvious from the list of currently ongoing clinical trials^10,13,14^.

A connection between Keap1 and autophagy has been documented through an interaction with the autophagy-related protein p62. In the absence of autophagy, p62 accumulates and competes with Nrf2 to bind to Keap1. Autophagy is a conserved process that ensures quality control of the cellular contents by their lysosomal degradation and recycle^15^. Autophagy consists of different steps defined as autophagy flux. Upon initiation of autophagosome formation by Beclin-1 and other key proteins, microtubule-associated protein light-chain 3 (LC3-I) is cleaved and then conjugated with phosphatidylethanolamine into LC3-II, directly binding to p62/SQSTM1^16^. p62 is an autophagy substrate that serves as a cargo receptor for autophagic degradation^16^. This protein is constantly degraded by autophagy, therefore, elevated p62 levels indicate dysfunctional autophagy. The whole process also requires autophagy-related (Atg) proteins, such as Atg3, Atg4, and Atg7. It has been shown that autophagy contributes to the onset and progression of a variety of diseases, including cancer^17^. In HNSCC, autophagy enhances the resistance towards nutrient deprivation and helps cells to survive in stressful environment, thereby driving tumorigenesis^18^. First hints exist suggesting failure to conventional radiochemotherapy to be co-determined by autophagy-mediated cell survival^18^. As Keap1/Nrf2 seems to play a prominent role in therapy resistance, it is worth noting that (i) Nrf2 controls p62 transcription, (ii) Keap1 participates in ubiquitin aggregate clearance via autophagy through association with LC3-II and p62, and (iii) p62 accumulation during autophagy impairment leads to inhibition of HR-mediated DSB repair^19–21^.

To identify the role of Keap1 in the radiosensitivity of HNSCC cells, we conducted a series of experiments exploring cytotoxicity and clonogenic survival, as well as DNA repair and autophagy upon Keap1 pharmacological inhibition. We identified Keap1 as critical determinant of cellular radiosensitivity and NHEJ-mediated DSB repair. Moreover, our data suggest autophagy to be induced in HNSCC cells when X-ray irradiation and Keap1 inhibition are applied simultaneously.

## Results

### Keap1 is overexpressed in head and neck cancers and its inhibition reduces clonogenic survival of HNSCC cells

**Fig. 1:**
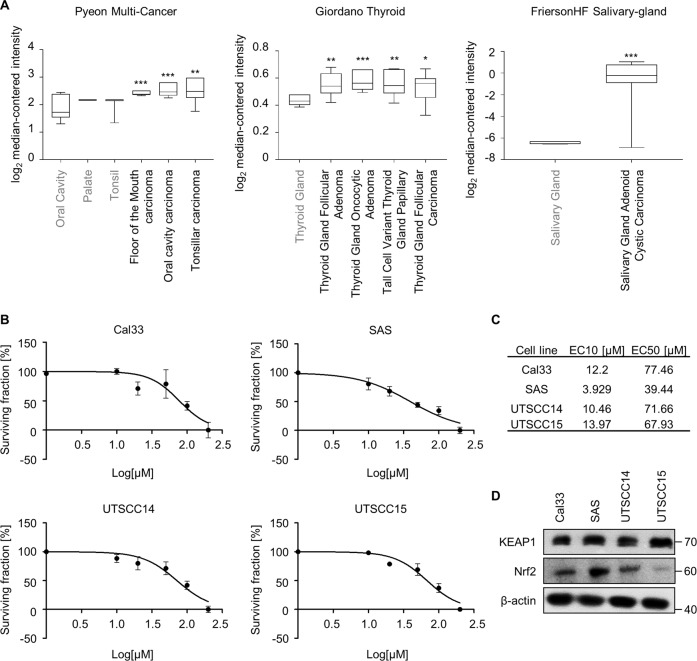
Elevated expression of Keap1 in HNSCC. **A** Oncomine database analyses of Keap1 mRNA expression in head and neck carcinomas (black) in comparison to corresponding normal tissue (gray). **B** Dose–response curves of ML334 (Keap1 inhibitor) in four head and neck squamous cell carcinoma cell line. **C** Table representing the EC10 and EC50 values extrapolated from the graphs shown in **B**. **D** Immunoblot of Keap1 and Nrf2 expression in indicated HNSCC cell lines. β-actin served as loading control.

A previously published whole-exome sequencing in a panel of HNSCC cell lines revealed a high mutational rate of the KEAP1 gene putatively resulting in alterations in protein characteristics^4^. Moreover, Keap1 was identified in a high-throughput screen in HNSCC cells as novel target critically involved in radioresistance and DNA repair processes^22^. In line with published data, we here corroborate Keap1 mRNA overexpression profiles in tumor versus corresponding normal tissues across multiple head and neck cancer databases (Pyeon Multi-Cancer, Giordano Thyroid, and FriersonHF Salivary-gland) using the Oncomine^TM^ platform^23^ (https://www.oncomine.org) (Fig. 1A).

Next, we explored the cytotoxic effects of a pharmacological Keap1 inhibition with the inhibitor ML334 in four Keap1- and Nrf2-expressing HNSCC cell lines (Fig. 1B–D). The four employed cell lines (Cal33, SAS, UTSCC14, UTSCC15) are HPV-negative, p53 mutated and all are squamous cell carcinomas originating from the tongue^4,24,25^. From this panel, SAS was the most sensitive cell line with the lowest EC10 and EC50, whereas Cal33 was as the most resistant cell line (Fig. 1C). Interestingly, we observed that Keap1 was similarly expressed in all the HNSCC cell lines (Fig. 1D), which is in line with the Oncomine data, while the expression level of Nrf2, a transcription factor regulated by Keap1, highly varied between the cell lines.

### Keap1 inhibition sensitizes HNSCC cells to X-rays and impairs DNA double-strand break repair cell line-dependently

**Fig. 2:**
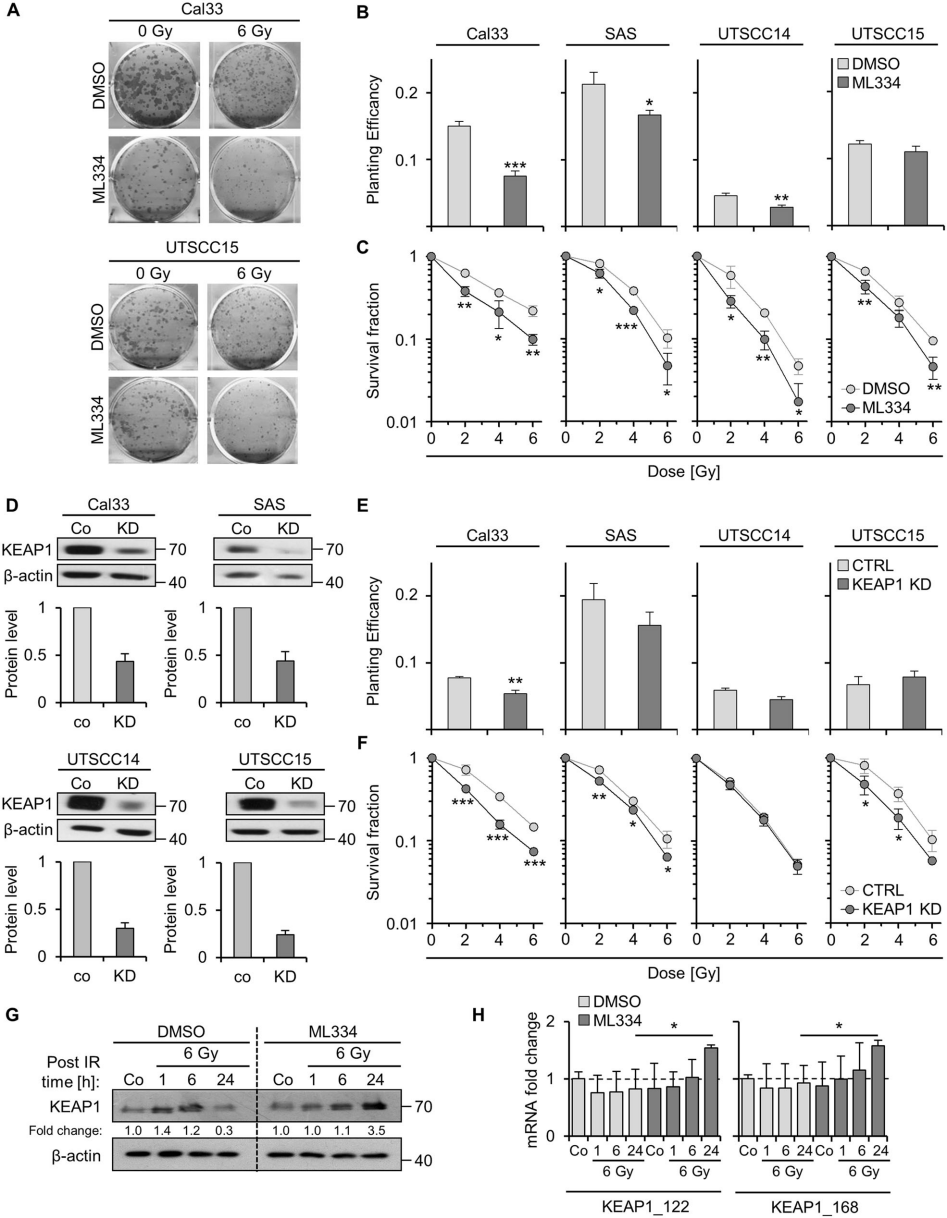
Keap1 inhibition sensitizes HNSCC cells to X-ray irradiation. **A** Representative images of colony forming assays upon indicated treatments. Clonogenic basal (**B**) and radiation (**C**) survival of four HNSCC cell cultures after 1-h pretreatment with ML344 (EC50) plus/minus X-rays (0–6 Gy). DMSO was used as control. **D** Immunoblotting of siRNA-mediated Keap1 knockdowns in four HNSCC cell lines. **E** Basal survival of HNSCC cell cultures after siRNA-mediated Keap1 knockdown (non-specific siRNA as control). **F** Clonogenic radiation survival of HNSCC cell cultures after siRNA-mediated Keap1 knockdown. **G** Immunoblotting of Keap1 kinetics after ML344 and X-ray exposure. β-actin served as loading control. Fold change quantifications are shown under the respective blots. **H** mRNA fold change of DMSO/ML334-treated Cal33 exposed to sham or 6 Gy X-ray. mRNA was extracted after 1, 6, and 24 h post irradiation. Data are presented as mean ± SD (*n* = 3; two-sided *t*-test; *P < 0.05, **P < 0.01, ***P < 0.001).

To study the function of Keap1 in radiation survival and DNA DSB repair, ML334 was applied to HNSCC cell lines one hour prior X-ray exposure. ML334 elicited significantly enhanced radiosensitivity in all tested HNSCC cell lines relative to controls (Fig. 2A, B). Knockdown of Keap1 using specific small-interfering RNA (siRNA) (Fig. 2D) showed a similar outcome (Fig. 2E, F), suggesting an essential role of Keap1 in cancer cell radiosensitivity. In order to address if the inhibitor was adequately specific, we employed cells previously shown to be radioprotected or insusceptible to Keap1 inhibition. These human lung and prostate cancer cell lines treated with similar concentrations to the EC10 and EC50 as applied for the HNSCC cell lines (10 µM and 60 µM, respectively). In line with the previous published data, Keap1 inhibition in lung or prostate cancer cells did not show any radiosensitizing effect (Suppl. Fig. 1A–F). Moreover, SKMES-1 cell line demonstrated a radioprotection upon 10 or 60 µM ML334. Interestingly, Keap1 protein levels showed to be induced already 1 h after X-ray irradiation, while ML334 seemed to delay this induction markedly (Fig. 2G). This was in line with the mRNA levels indicating an increase of KEAP1 mRNA 24 h after ML334/X-ray irradiation (Fig. 2H).

**Fig. 3:**
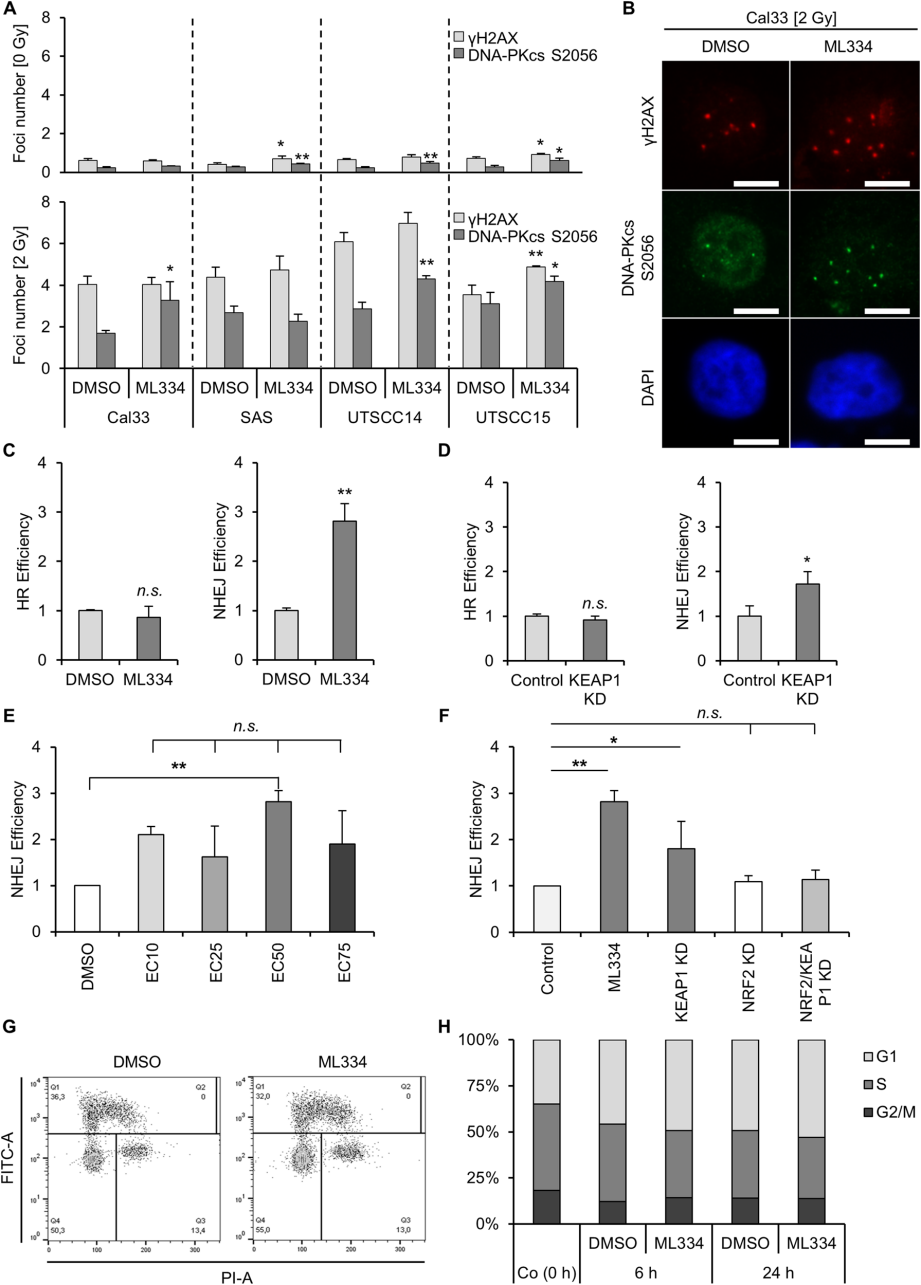
Keap1 regulates non-homologous end joining. **A** Effect of Keap1 inhibition on residual γH2AX (light gray) and DNA-PKcs S2056 (dark gray) foci in HNSCC cell lines after sham or 2-Gy irradiation. **B** Representative immunofluorescence images of residual γH2AX (red) and DNA-PKcs S2056 (green) after indicated treatments (bar, 10 µm). **C** GFP-based reporter assays for homologous recombination (HR) and non-homologous end joining (NHEJ). Cal33 cells stably transfected with DRGFP or pimEJ5GFP recombinant plasmids were treated with ML334 (EC50) or DMSO. The number of GFP-positive cells was analyzed by flow cytometry. **D** Cal33 cells stably transfected with DRGFP or pimEJ5GFP recombinant plasmids were depleted of Keap1 by siRNA-mediated knockdown. The number of GFP-positive cells was analyzed by FACS. **E** Cal33 cells stably transfected with pimEJ5GFP recombinant plasmids were treated with EC10, EC25, EC50 and EC75 of ML334 (DMSO used as control). The number of GFP-positive cells was analyzed by FACS. **F** Cal33 cells stably transfected with pimEJ5GFP recombinant plasmids were depleted of Keap1 and NRF2 by knockdown. Results are compared to ML344 (EC50). The number of GFP-positive cells was analyzed by flow cytometry. **G** Representative dot plot figures of cell cycle distribution of UTSCC14 cells upon Keap1 inhibition. **H** Flow cytometry-based quantification of cell cycle distribution at 6 and 24 h post 6 Gy X-ray irradiation and ML334. Data are presented as mean ± SD (*n* = 3; two-sided *t*-test; **P < 0.01, ***P < 0.001; n.s., not significant (*P* ≥ 0.05)).

In accordance with the enhanced radiosensitivity already at 2 Gy, the repair of DSB was investigated upon ML334 and 2 Gy. ML334 elicited significantly elevated γH2AX and/or DNA-PKcs S2056 foci numbers in the three out of four 2-Gy irradiated HNSCC cell lines (Fig. 3A, B and Suppl. Fig. 2A–C). We observed similar trends for γH2AX and DNA-PKcs S2056 foci formation and resolution upon Keap1 siRNA knockdown compared with controls (Suppl. Fig. 2D, E). Moreover, as reactive oxygen species (ROS) are associated with radiogenic DNA damage, we measured ROS levels in ML344-treated and untreated cells and found no significant difference upon X-ray irradiation (Suppl. Fig. 2F).

Collectively, our results suggested that Keap1 plays an essential role in the radiosensitivity and repair of radiogenic DSB in HNSCC cells.

### Keap1 regulates NHEJ in an Nrf2-dependent manner

To further address the function of Keap1 in DSB repair, we conducted DNA repair reporter assays to measure HR and NHEJ activities in Cal33 cells (Fig. 3C, D). HR activity remained unaffected, whereas NHEJ activity was significantly enhanced upon both ML334 and siRNA-mediated Keap1 depletion by approximately 3-fold and 2-fold, respectively (Fig. 3C, D). The induction of NHEJ activity by different concentrations of ML334 showed similar values (Fig. 3E).

However, as Keap1 is an upstream regulator of Nrf2, inhibition of Keap1 might be influenced by Nrf2. Hence, we performed single and double knockdown of Keap1 and Nrf2. We found a normalization of the NHEJ activity upon both single Nrf2 and simultaneous Nrf2/Keap1 depletion indicative of a dominant role of Nrf2 over Keap1 regarding this DNA repair process (Fig. 3F and Suppl. Fig. 2G).

To exclude a strong impact of cell cycle alterations under Keap1 inhibition, cell cycle kinetics of UTSCC14 cells at 0, 6, and 24 h post irradiation were conducted. The data revealed no significant alteration upon Keap1 inhibition in unirradiated and irradiated cells (Fig. 3G, H). This result suggested that the increase in NHEJ activity is unassociated with cell cycle arrest. Taken together, our data indicate that Keap1 critically modulates NHEJ in an Nrf2-dependent and cell cycle-independent manner.

### Keap1 influences DNA-PKcs phosphorylation and Rad50 levels

**Fig. 4:**
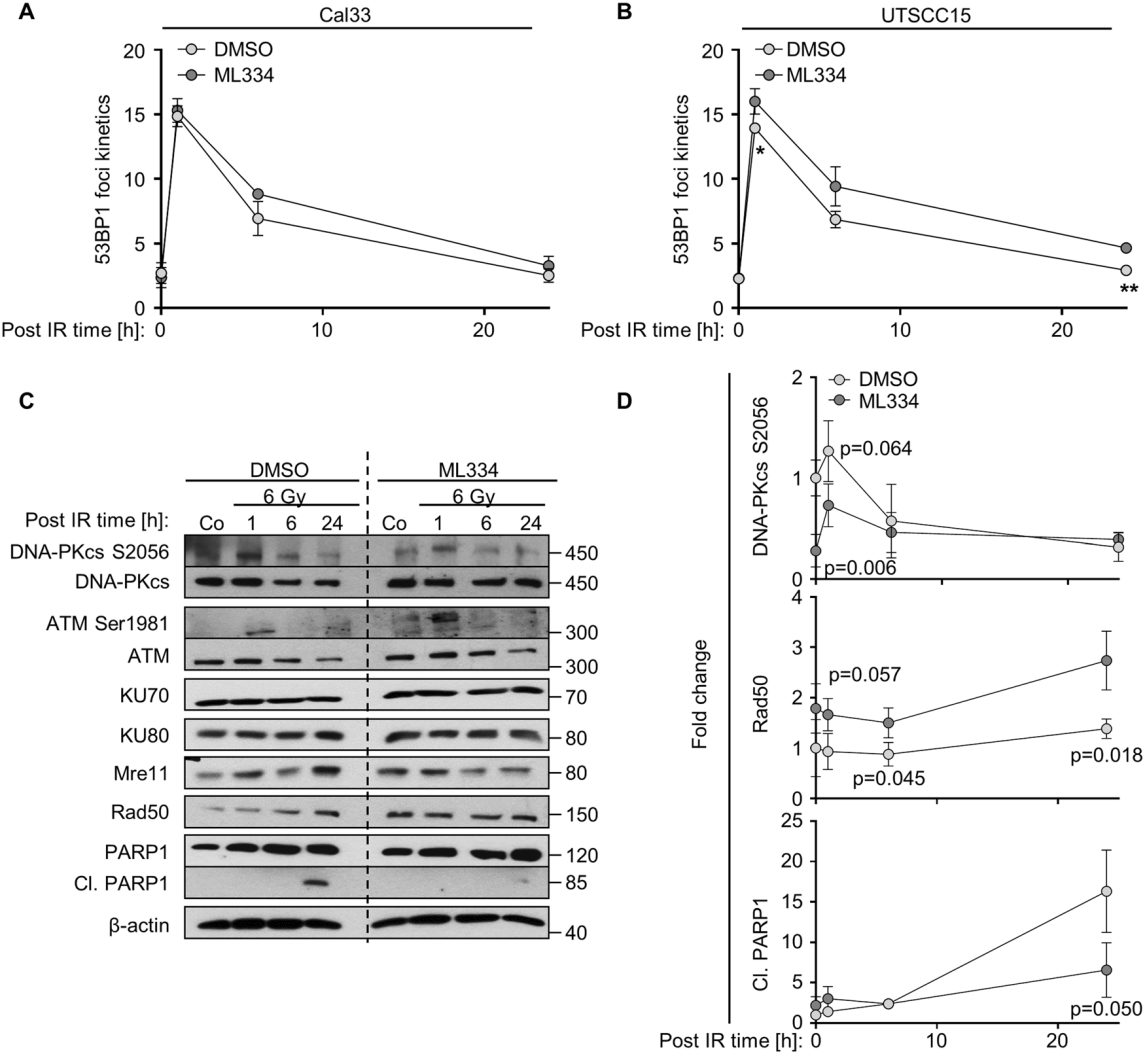
NHEJ kinetics after Keap1 inhibition. **A**, **B** 53BP1 foci kinetics at different time points (1, 6, and 24 h) post 2 Gy X-ray exposure and ML344 in two HNSCC cell lines. **C** Immunoblots and **D** densitometries in whole cell lysates from ML334-treated/6-Gy irradiated Cal33 cells showing total and/or phosphorylated forms of DNA-PKcs, ATM, Ku70, Ku80, Mre11, Rad50, and PARP1. Based on densitometries, phosphorylation levels were calculated relative to the total amount of the respective protein. β-actin served as loading control. Data are presented as mean ± SD (*n* = 3; two-sided *t*-test; *P < 0.05, **P < 0.01).

To study the underlying mechanism of the impact of Keap1 on NHEJ, we investigated the foci kinetics of 53BP1 associated with NHEJ in Cal33 and UTSCC15 cells. A trend could be identified showing an increase in intermediate (1 and 6 h post irradiation) and residual 53BP1 foci (24 h post irradiation) numbers in ML334-treated versus DMSO-treated cells (Fig. 4A, B). This increment of 53BP1 foci formation upon ML334-mediated Keap1 inhibition provides a first hint of an involvement of Keap1 in the repair of radiogenic DSB. In addition, the phosphorylation and the expression of other key associated proteins i.e., DNA-PKcs, ATM, Ku70, Ku80, MRN complex (classical NHEJ) PARP1, cleaved-PARP1 (alternative NHEJ) revealed changes to borderline or non-significant differences between ML334-treated and untreated cells (Fig. 4C). Overall, we found that Keap1 inhibition causes a defect in the radiogenic hyperphosphorylation of both DNA-PKcs at S2056, while Rad50 expression levels significantly increased (Fig. 4C, D and Suppl. Fig. 3). In contrast to a restrained DNA-PKcs phosphorylation, the phosphorylation of ATM at S1981 was moderately increased in ML334/X-ray-treated Cal33 cells (Suppl. Fig. 3B). To explore the dependency of the NHEJ hyperactivity upon Keap1 inhibition on ATM, we simultaneously treated Cal33 cells with ML334 plus the ATM inhibitor KU55933 or KU55933 alone and discovered significant NHEJ activity reduction by KU55933 alone relative to control but no significant difference upon ML334/KU55933 compared with control (Suppl. Fig. 3H). This implies that the induction of NHEJ by Keap1 inhibition does not directly involve ATM. Altogether, these results indicate an essential role of Keap1 in NHEJ.

### LC3 nuclear puncta accumulation depends on Keap1

**Fig. 5:**
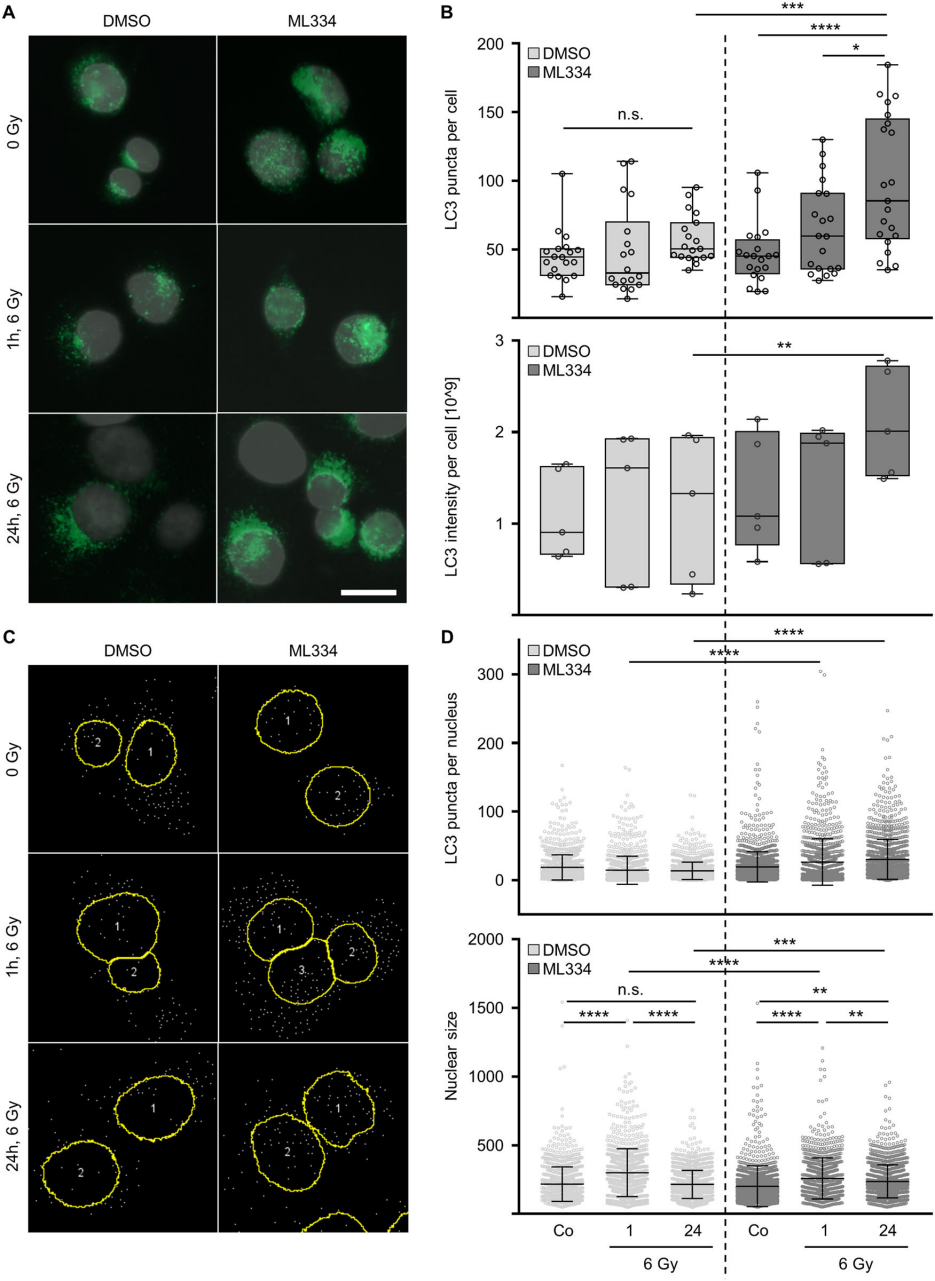
ML344 impacts on total and nuclear LC3 puncta levels. **A** Representative immunofluorescence images of LC3 puncta (green) and DAPI (gray) after 1 and 24 h post 6-Gy irradiation plus ML334 treatment (DMSO served as control) (bar, 20 µm). **B** Puncta quantification and intensity analysis of cells using Fiji software. **C** Representative analyzed images of LC3 maxima inside the nuclear mask after 1 and 24 h post 6-Gy irradiation/ML334. **D** Nuclear LC3 maxima quantification and nuclear area analysis of cells using Fiji software. Data are represented as mean ± SD (*n* = 3; one-way Anova, multi-comparison analysis; *P < 0.05, **P < 0.01, ***P < 0.001, ****P < 0.0001; n.s., not significant (*P* ≥ 0.05)).

Based on enhanced radiosensitivity and a documented linkage between Keap1 and autophagy-related proteins, we elucidated Keap1 inhibition in the context of autophagy. LC3 immunofluorescent kinetics revealed significant changes in LC3 intensity per cell and number of LC3 puncta per cell in cells treated with ML334 and X-ray irradiation relative to controls (Fig. 5A, B). Exposure of cells with DMSO and irradiation did not significantly affect the LC3 puncta per cell. In addition, nuclear LC3 puncta were analyzed and showed a significantly larger number of LC3 puncta accumulated in the nucleus of ML334-treated and irradiated cells relative to controls (Fig. 5C). In parallel, nucleus size remained enlarged in ML334-treated and irradiated at 1 h and 24 h post irradiation compared with controls (Fig. 5D).

### Keap1 influences autophagy by regulating p62 and Nbs1

**Fig. 6:**
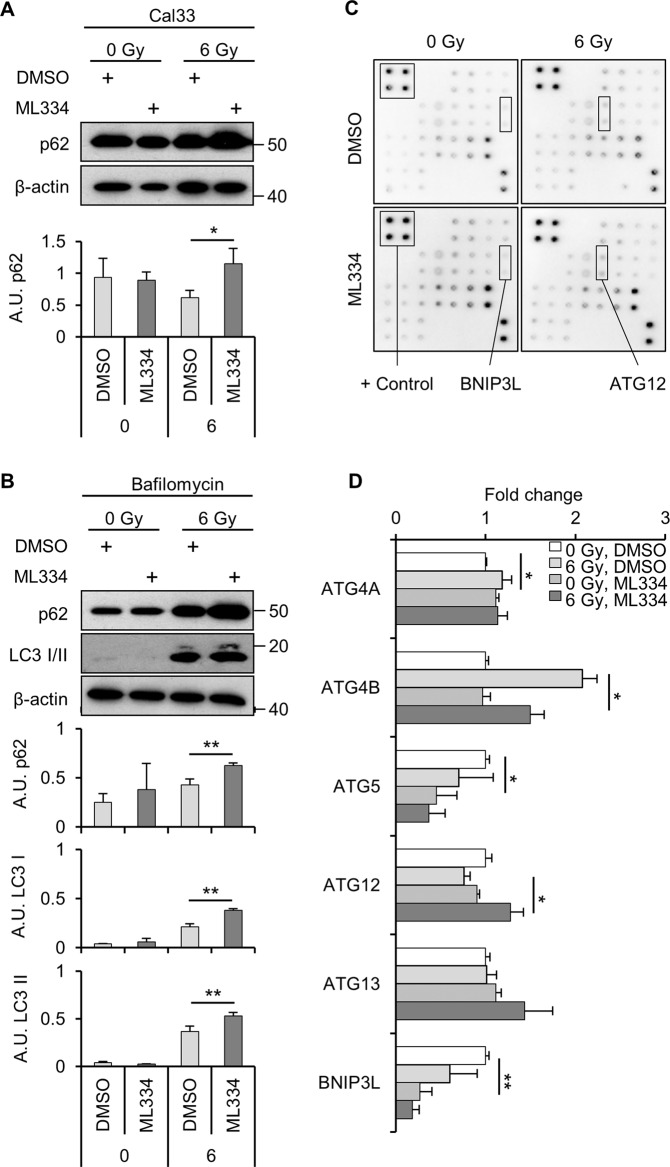
Keap1 impacts on autophagy-related proteins. **A** Immunoblot and densitometries pf p62 in whole cell lysates from ML334/6-Gy irradiated Cal33 cells. β-actin served as loading control. **B** Immunoblot on whole cell lysates from cells pretreated with Bafilomycin (inhibitor of the autophagosome–lysosome fusion), ML334 and 6-Gy X-rays. **C** Representative images of autophagy microarray. **D** Array results of 6 Gy/ML344-treated Cal33 cells. Data represents fold changes relative to controls (0 Gy, DMSO). Data are represented as mean ± SD (for array *N* = 2 and *n* = 2; for western blots *n* = 3; two-sided *t*-test; *P < 0.05, **P < 0.01).

To further investigate a putative connection between Keap1 and autophagy, we evaluated p62 expression levels upon Keap1 inhibition. p62 protein levels were increased after ML334 treatment in combination of X-ray (Fig. 6A). Confirmatory data were generated by using the autophagosome–lysosome fusion blocker bafilomycin. We observed an increase of p62, LC3-I and LC3-II protein levels in Keap1 inhibited and irradiated Cal33 cells relative to controls (Fig. 6B). These findings suggest an impact of Keap1 on autophagy in irradiated HNSCC cells.

Using an autophagy array, additional effects of a combined ML344/irradiation treatment became apparent (Fig. 6C, D, Suppl. Fig. 4A and Suppl. Table 1). ML334 already elicited some effects on autophagy-related proteins (Fig. 6D) detectable as ATG4A induction and ATG5 and BNIP3L downregulation. Combined ML344 and irradiation significantly reduced ATG4B and increased ATG12, two key regulators of autophagy (Fig. 6A, B). In line with this, database analysis^26,27^ (https://www.cbioportal.org/, https://reactome.org/) of Keap1 mRNA in HNSCC (TCGA, PanCancer Atlas) demonstrated a positive correlation with ATG4B and ATG4D, as well as a negative correlation with ATG12 (Suppl. Fig. 4B, C). Furthermore, by using Cytoscape^28^, we observed a predicted interplay between key autophagy-regulating proteins and Keap1 (Suppl. Fig. 4D). Taken together, these data indicate an association between Keap1 and autophagy-related proteins of the Atg protein family.

## Discussion

Treatment resistance remains one of the major challenges for cancer patient survival. To safeguard survival, cells employ multiple complex mechanisms. The Keap1/Nrf2 system has been shown to be, on the one hand, critical for cell survival and, on the other hand, dysregulated in cancer. Based on genetic data from our group and others^4,7^, we sought to pharmacologically deactivate Keap1 in HNSCC cells and found that Keap1 inhibition (i) enhances HNSCC cell radiosensitivity, (ii) elevates the activity of NHEJ-related DNA repair in a Nrf2-dependent manner, (iii) impairs DSB repair through delayed phosphorylation of DNA-PKcs, and (iv) elicits autophagy and increased p62 levels when combined with X-ray irradiation.

In line with others^7^, we show that Keap1 mRNA is overexpressed in head and neck cancers. As published data claims deactivating mutations causative for optimized Nrf2-mediated surveillance of the oxidative stress system, pharmacological inhibition seems effective but requires further investigation. Reports consistently state protective effects for cells when Keap1 is deactivated^29–31^. Consequently, cells exposed to ionizing radiation under Keap1 inhibition are supposed to show elevated survival due to prosurvival Nrf2-related effects. While studies in human lung fibroblasts^32^, human hepatocellular carcinoma cells^33^, and human prostate cancer cells^34^ support this notion, our own studies in HNSCC cell lines show contradictory findings. In a previously published siRNA-mediated knockdown screen and in the present study, we demonstrate that Keap1 inhibition significantly enhances HNSCC cell radiosensitivity.

Based on our observations, we here show first data of our efforts to identify putative underlying mechanisms for radiosensitization through Keap1 inhibition. Of interest in this context are recently published data demonstrating the two main DNA repair processes, i.e., HR and NHEJ, to be co-regulated by both nuclear and cytoplasmic cues and by autophagy^35^. Moreover, it is discussed that autophagy may impact DSB repair pathways and genome integrity through the degradation of nuclear components^9^. Currently, it remains to be clarified whether an induction^36^ or loss of autophagy impairs DNA damage repair^19,37–39^. It seems highly likely that this autophagy/DNA repair interplay is cell type- and context-dependent. Our data suggest induced NHEJ activity in combination with perturbed DNA-PKcs functionality without changes in HR, as well as an induction of autophagy. A putative linker protein is p62. It has been shown that autophagy deficiency causes inefficient HR-mediated DSB repair^19,37,40^. Proposed mechanisms are increased proteasomal degradation of the Checkpoint kinase 1, an important coordinator of cell cycle and HR^37^ and increased p62 levels^19,40^. Upon ML334, HNSCC cells exhibit p62 protein induction and delayed auto-phosphorylation of DNA-PKcs (S2056). Additional supportive data of this study document elevated nuclear accumulation of LC3 puncta after X-ray exposure. It is known that acetylation of LC3 causes its nuclear translocation, while deacetylation upon starvation stimulates nuclear-cytoplasmic shuttling. Further, the nuclear pool of LC3 has a specific function in priming starved cells for autophagy induction underpinning our finding that autophagy induction might be part of the enhanced cell killing upon Keap1 inhibition. In line, recent studies showed nuclear LC3 accumulation to be associated with cell death in colorectal cancer cells and human oocytes^41,42^. Regarding altered levels of Rad50, cleavage PARP1, pATM S1981, Ku80 and MRE11, we believe that the detected changes occur through a cellular attempt to compensate between NHEJ and HR repair processes. Further investigations are warranted.

In summary, our results reveal a critical function of the Keap1 protein in regulating the radiosensitivity of HNSCC cells through modulating NHEJ-mediated DSB repair and autophagy in a Nrf2-dependent manner. According to the concept of a synthetic lethal relationship between autophagy and NHEJ, our findings further add to the complex cross talk between putative pro- and anti-survival mechanisms and how these might be therapeutically exploitable.

## Materials and methods

### Cell culture

HNSCC cell lines (Cal33, SAS, UTSCC14, UTSCC15) were kindly provided by R. Grenman (Turku University Central Hospital, Finland). Cal33-DRGFP and Cal33-pimEJ5GFP were kindly provided by K. Borgmann (University-Medical Center Hamburg-Eppendorf, Germany). A549 and SKMES-1 cell purchased from American-Type Culture Collection (Bethesda, MD, USA). PC3 cells were kindly provided by A. Dubrovska (Technische Universität Dresden, Germany). Cells were cultured in Dulbecco’s modified Eagle’s medium containing glutamax-I (from AppliChem), 10% fetal calf serum and 1% non-essential amino acids (all PAA Laboratories) at 37 °C in a humidified atmosphere with 8.5% CO_2_. All cell lines were authenticated using a STR DNA profiling and tested negative for mycoplasma contamination.

### siRNA transfection

SignalSilence® KEAP1 siRNA II #5289 was purchased from Cell Signaling. Nrf2 siRNA (5ʹ-GGAGCUAUUAUCCAUUCCUTT-3ʹ) (16708) and Silencer Negative Control siRNA (5ʹ-AAAACAGUUGCGCAGCCUGAAtt-3ʹ) (AM4635) were obtained from Ambion. siRNA transfection was carried out as previously published^43^. Briefly, 24 h after plating, cells were incubated with 20 nM of siRNA pre-mixed with 4 µl oligofectamine in serum-free Opti-MEM (Invitrogen) for 8 h. Next, Opti-MEM supplemented with 10% foetal calf serum was added to the cells. Twenty-four hours after the transfection, cells were used for clonogenic survival assay and DSB analysis.

### Inhibitor treatment and irradiation

Keap1 inhibitor ML334 (Axon Medchem) was applied at effective concentrations EC50 (DMSO as control), ATM inhibitor KU55933 (Calbiochem) at 10 µM and bafilomicin (Biomol) at 10 nM. Irradiation was performed after 1 h of inhibitor incubation at room temperature using single doses of 200-kV X-rays (Yxlon Y.TU 320; Yxlon; dose rate ~1.3 Gy/min at 20 mA) filtered with 0.5 mm Cu as described previously^44^. Dosimetry for quality assurance was performed using a Duplex dosimeter (PTW Freiburg) prior to irradiation.

### Colony formation assay

Cells transfected with siRNA were plated on 6-well plates and irradiated 24 h after seeding as published^44^. On the next day, ML334 was added one hour before irradiation and the cells were left to form colonies at 37 °C in a humidified atmosphere with 8.5% CO_2_. After a cell line-dependent growth time, cells were fixed with 80% ethanol and stained with Coomassie Brilliant Blue dye. Colonies consisting of >50 cells were counted under stereomicroscope.

### Antibodies and reagents

Antibodies against DNA-PKcs (#4602), Ku80 (#2180), Mre11 (#4895), Nbs1 (#3002), PARP1 (#9542), Rad50 (#3427), LC3-B (#2775S) and SQSTM1/p62 (#5114S) were from Cell Signaling; β-actin (#A1978) was from Sigma-Aldrich; γH2AX S139 (#05–636) was from Upstate and Ku70 (#ab3114), ATM (#2873), DNA-PKcs S2056 (#ab18192) were from Abcam; 53BP1 was from Novus Biologicals (Cambridge, UK; #NB100–904)); ATM S1981 (#200–301–400) was from Rockland; NRF2 (#sc-365949) was from Santa Cruz; BrdU (#347580) was from BD; horseradish peroxidase-conjugated donkey anti-rabbit (#NA-934) and sheep anti-mouse (#NA-931) secondary antibodies were from GE Healthcare; Alexa Fluor 594 anti-mouse (#A11032), Alexa Fluor 594 anti-rabbit (#A11037), Alexa Fluor 488 anti-mouse (#A11029) and Alexa Fluor 488 anti-rabbit (#A11034; Invitrogen).

### Western blotting

Cell lysis was performed using RIPA lysis buffer supplemented with protease inhibitor (Complete protease inhibitor cocktail from Roche) and phosphatase inhibitors (Na3VO4 and NaF from Sigma) as published^43^. The lysates were incubated for 30 min on ice prior to processing. Protein concentration was determined with bicinchoninic acid (BCA) assay Protein Assay Kit (Pierce). Proteins were separated using SDS polyacrylamide gel electrophoresis and transferred to nitrocellulose membranes. Next, the membranes were blocked using 5% non-fat dry milk diluted in PBST and incubated with primary antibodies overnight at 4 °C. Afterwards, the membranes were probed with secondary antibodies conjugated with horseradish peroxidase for 1.5 h at room temperature Chemiluminescent detection was preformed using ECL™ Prime Western Blotting System (Sigma-Aldrich).

### Quantitative real-time PCR

Total RNA from DMSO and ML334-treated cells, exposed to 6 Gy X-ray or left unirradiated, was extracted using the NucleoSpin RNA II kit (Macherey-Nagel) and following manufacturer’s protocol. Superscript III Reverse Transcriptase (Invitrogen) was employed for reverse transcription according to the manufacturer’s protocol. Target gene sequences were amplified and measured using TB Green Premix Ex Taq™ (Tli RNase H Plus) qPCR kit (Takara, Kusatsu, Japan) and StepOnePlus Real-Time PCR System (Thermo Fischer Scientific, Waltham, MA, USA). The primer sequences are: TBP-Fw: ACGAACCACGGCACTGATTT; TBP-Rev: ACTTCACATCACAGCTCCCC; ACTB-Fw: GAGAAAATCTGGCACCACACC; ACTB-Rev: GGATAGCACAGCCTGGATAGCA; KEAP1_122_Fw: GATGGCCACATCTATGCCGT; KEAP1_122_Rev: CCGATCCTTCGTGTCAGCAT; KEAP1_168_Fw: ATGCATTTTGGGGAGGTGGC; KEAP1_168_Rev: GACGTAGAACCGTCGCTGT.

### Foci assay

For DSB analysis, 50,000 cells were plated on glass coverslips and treated with ML334 (DMSO as a control) followed by 2 Gy X-rays irradiation on the next day. Later, cells were fixed with 3% formaldehyde/phosphate-buffered saline (Merck) at 1, 6, and 24 h after irradiation (non-irradiated cells as a control). Thereafter, cells were permeabilized with 0.25% Triton-X-100/phosphate-buffered saline (Roth) and immunostained with antibodies against γH2AX, DNA-PKcs S2056 and 53BP1 as published^43^. Finally, coverslips were mounted in Vectashield/4′-6-diamidino-2-phenylindole (Alexis) mounting medium. Foci numbers were evaluated with an AxioImager A1 plus fluorescence microscope (Carl Zeiss) under a ×40 objective. Representative images were obtained using AxioImager M1 (Carl Zeiss).

### LC3 immunostaining

Fifty-thousand cells were seeded on glass coverslips and incubated for 24 h. Afterwards, ML334 was applied in cell line-dependent effective concentrations. One hour after the treatment, cells were irradiated with 6 Gy X-rays and fixed at different time points (1 and 24 h) with 100% MetOH at −20 °C for 15 min. Fixed and permeabilized cells were washed with PBS, blocked with 5% BSA/PBS for 30 min and incubated with primary antibodies overnight at 4 °C as previously described^44^. On the next step, secondary antibodies were applied for 1 h at room temperature in the darkness. Finally, coverslips were stained with DAPI and analyzed with confocal laser scanning microscopy. Data analysis was performed using Fiji ImageJ and more than 150 cells were analyzed per sample.

### DRGFP and pimEJ5GFP-based chromosomal break reporter assay

Cal33-DRGFP and Cal33-pimEJ5GFP cells^45^ were transfected with Keap1, Nrf2 or scramble, non-specific control siRNAs as published^43^. After 8 h, cells underwent the second transfection with SceI-expressing plasmid pcDNA3BMyc-NLS-ISceI (I-SceI) or control pEGFP-N1 (pN1) plasmid using lipofectamine 2000 pre-diluted in Opti-MEM (Invitrogen) according to the manufacturer’s instructions. Four hours later, medium was replaced with growth medium. In the experiments with ML334, cells were first transfected with the pN1 and I-SceI plasmids. Five hours after transfection, fresh medium was added together with the inhibitor. After 48 h, cells were trypsinized and analyzed using flow cytometer FACS Celesta (BD Biosciences) and FlowJo software (version 7.6.2).

### Cell cycle analysis

Keap1 depleted UTSCC14 cells were irradiated with 6 Gy X-rays and cultured for 6 and 24 h later on (non-specific RNA as control). Then, cells were incubated with 10 mM BrdU (BD Biosciences) for 10 min and harvested using trypsin/EDTA solution. Thereafter, cell suspension was fixed with ice-cold 80% ethanol for 10 min and incubated for 10 min with 0.01% RNase A (Sigma-Aldrich) followed by 30 min treatment with 2 N HCl (Sigma-Aldrich) and 0.5% Triton-X-100/PBS (Roth). Subsequently, mouse anti-BrdU antibodies and propidium iodide (Sigma-Aldrich) was added for the analysis of BrdU incorporation and total DNA content. As previously published^46^, Cell cycle distribution was determined using a FACS Celesta (BD Biosciences) with FlowJo software (version 7.6.2).

### Autophagy microarray

RayBio® C-Series Human Autophagy Array 1 Kit (AAH-ATG-1-8) was used to identify changes in the expression of autophagy-related proteins upon ML334. Cal33 cells exposed to ML334 1 h prior to 6 Gy X-ray irradiation were lyzed and processed according to the manufacturer’s instructions (https://www.raybiotech.com/files/manual/Antibody-Array/AAH-ATG-1.pdf) on the next day. Protein concentration was measured using BCA kit. Fusion FX (VILBER) detection machine and Fiji software were used to calculate protein expressions.

### Data analysis

Means ± standard deviation (SD) of at least three independent experiments were calculated with reference to controls defined in total numbers or 1.0. For statistical significance, two-sided Student’s *t*-test was performed using Microsoft Excel 2003. *P*-value of <0.05 was considered statistically significant. For maxima analysis one-way ANOVA followed by multiple comparison analysis was performed by GraphPad Prism7.

## Supplementary information

Supplemental Material_unmarked

Suppl. Fig. 1

Suppl. Fig. 2

Suppl. Fig. 3

Suppl. Fig. 4

Supplementary Table
