# Supplementary material for: Keap1 inhibition sensitizes head and neck squamous cell carcinoma cells to ionizing radiation via impaired non-homologous end joining and induced autophagy
Source: Cell Death Dis. 2020 Oct 21;11(10):887. doi: 10.1038/s41419-020-03100-w (PMC7578798; doi:10.1038/s41419-020-03100-w)
Supplement: Supplementary file 1 — Supplemental Material_unmarked [file 41419_2020_3100_MOESM1_ESM.doc]

**Keap1 inhibition sensitizes head and neck squamous cell carcinoma cells to ionizing radiation via impaired non-homologous end joining and induced autophagy**

Sara Sofia Deville, Susanne Luft, Maria Kaufmann & Nils Cordes

**Supplementary Material**

**Supplementary Fig. 1| Keap1 inhibition in lung and prostate cancer cell lines.** Clonogenic basal and radiation survival of two lung cancer cell cultures (**A, B,** A549 and **C, D,** SKMES-1) and one prostate cancer cell line (**E, F,** PC3) after ML344 (10 µM or 60 µM) plus/minus X-rays (0-6 Gy) treatment. DMSO was used as control. Data are presented as mean ± SD (n = 3; two-sided t-test; *P<0.05).

**Supplementary Fig. 2| Keap1 knockdown impairs DSB repair. A**, Profile analysis of γH2AX and DNA-PKcs S2056 foci in Cal33 cells exposed to 2-Gy irradiation. Profiles were generated using ImageJ. **B**, and **C**, Histograms showing number of cells presenting either γH2AX (**B**) or DNA-PKcs S2056 (**C**) foci in Cal33 cells upon 2-Gy X-ray exposure. **D**, Representative immunofluorescence images of residual γH2AX (red) and DNA-PKcs S2056 (green) after ML334/2-Gy treatment (DMSO as control) (bar, 10 µm). **E**, Effect of Keap1 knockdown on residual γH2AX (light grey) and DNA-PKcs S2056 (dark grey) foci in HNSCC cell lines after 2-Gy irradiation (non-specific siRNA as control). **F**, ROS levels of cells treated ML334/6 Gy (DMSO as control). Analysis were performed 2 h post irradiation. **G**, KEAP1 and NRF2 knockdown in Cal33-pimEJ5GFP cells. Data are presented as mean ± SD (n = 3; two-sided t-test; *P<0.05, **P<0.01, ***P<0.001); n.s., not significant (P≥0.05)).

**Supplementary Fig. 3| Keap1 and DSB repair proteins.** Densitometries of **A**, total DNA-PKcs, **B**, phosphorylated ATM S1981, **C**, ATM, **D**, Ku70, **E**, Ku80, **F**, Mre11 and **G**, PARP1 total proteins. **H**, Cal33 cells stably transfected with pimEJ5GFP recombinant plasmids were treated with ATM inhibitor (ATMi) plus/minus ML334 (DMSO as control). Data are presented as mean ± SD (n = 3; two-sided t-test; *P<0.05, **P<0.01, ***P<0.001); n.s., not significant (P≥0.05)).

**Supplementary Fig. 4| Keap1 alters expression of autophagy-related proteins. A**, Densitometries of all autophagy-related proteins present in the array. **B**, Autophagy-related genes obtained from Reactome database were employed for identification of mRNA Keap1 correlated genes accessed from cBioPortal. The used datasets were from HNSCC (TCGA, PanCancer Atlas). The plot shows on the x-axis the Pearson’s correlation of the mRNA for autophagy-related genes and Keap1 mRNA. On the y-axis, the –Log10(p-Value) of these correlations are plotted. The higher the dots are located, the more significant is the correlation. In red are marked some important autophagy proteins which are negatively correlated to Keap1 mRNA expression. While in green are shown the positive correlated protein of interest. **C**, Correlations of fundamental genes for autophagy and Keap1 obtained from cBioPortal. On the x-axis is shown the Log2 of the mRNA expression of Keap1 and on the y-axis the Log2 of the selected genes mRNA expression. **D**, Cystoscape predicted clustering from Keap1 and p62 input. Data are presented as mean ± SD (n = 3; two-sided t-test; *P<0.05, **P<0.01, ***P<0.001).

**Supplement Table 1. Data from two independent trials of the autophagy array.**
