# Supplementary figures and images for: Keap1 inhibition sensitizes head and neck squamous cell carcinoma cells to ionizing radiation via impaired non-homologous end joining and induced autophagy
Source: Cell Death Dis. 2020 Oct 21;11(10):887. doi: 10.1038/s41419-020-03100-w (PMC7578798; doi:10.1038/s41419-020-03100-w)

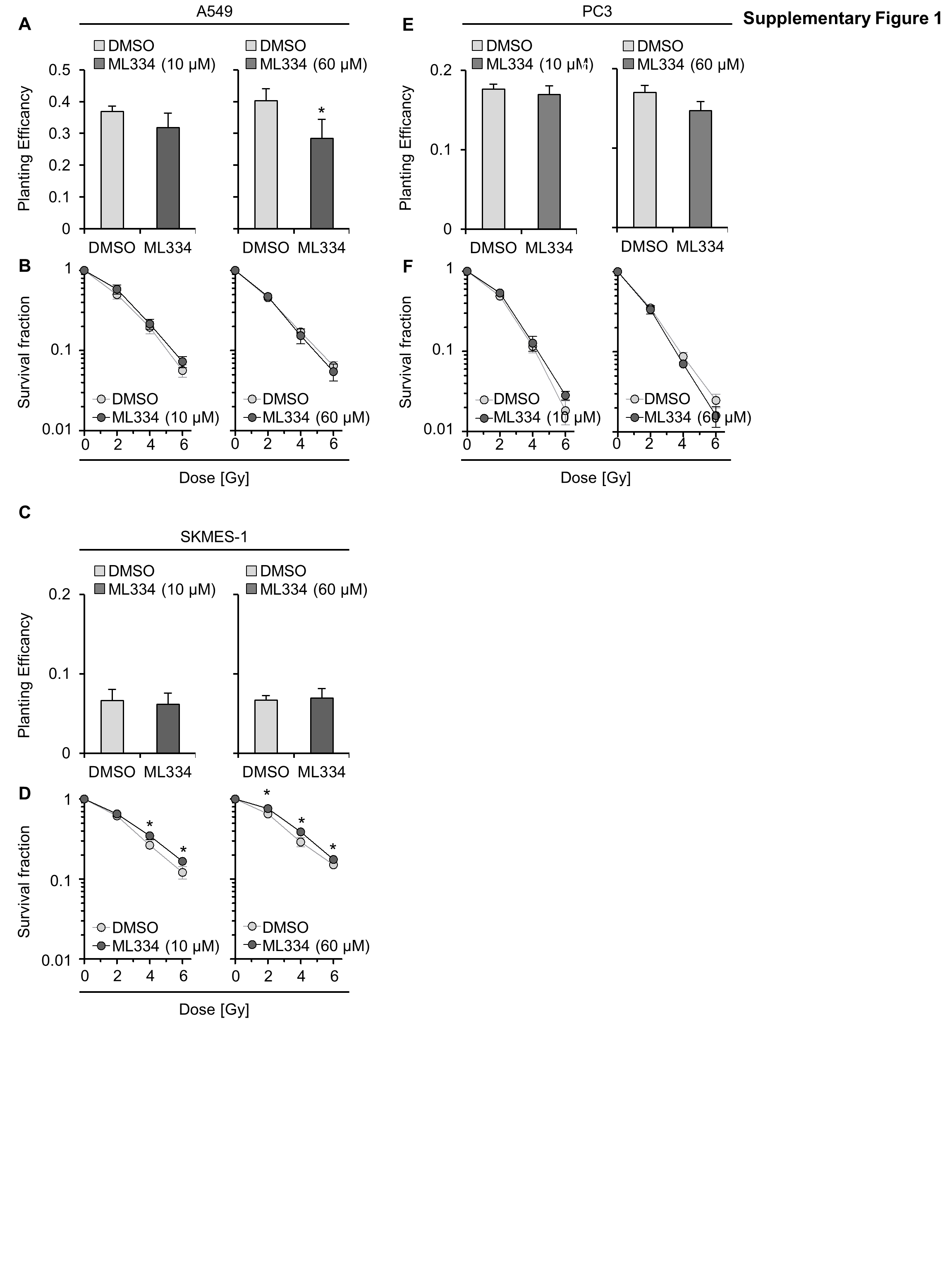

Supplement: Supplementary file 2 — Suppl. Fig. 1 [file 41419_2020_3100_MOESM2_ESM.tif]

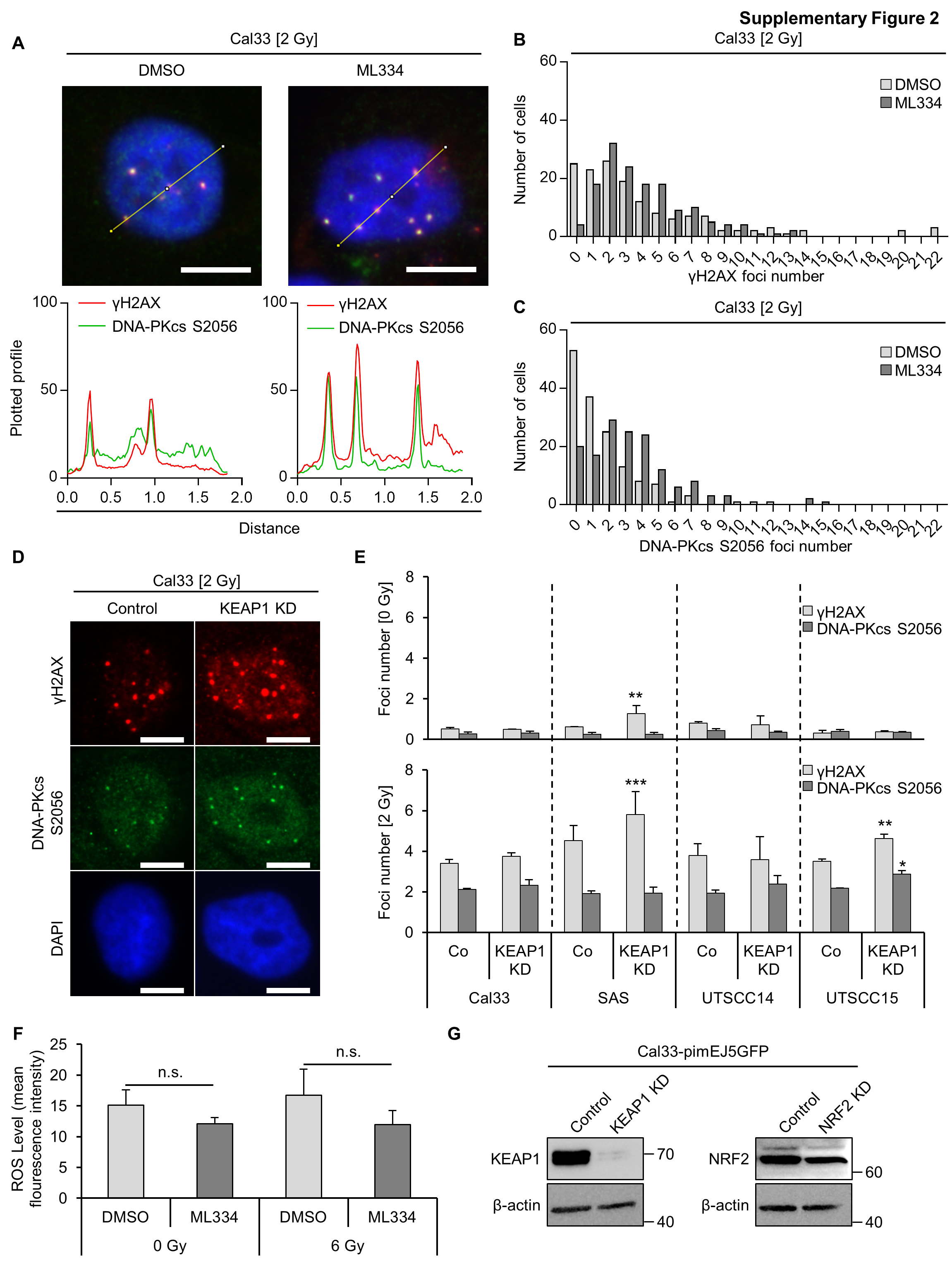

Supplement: Supplementary file 3 — Suppl. Fig. 2 [file 41419_2020_3100_MOESM3_ESM.tif]

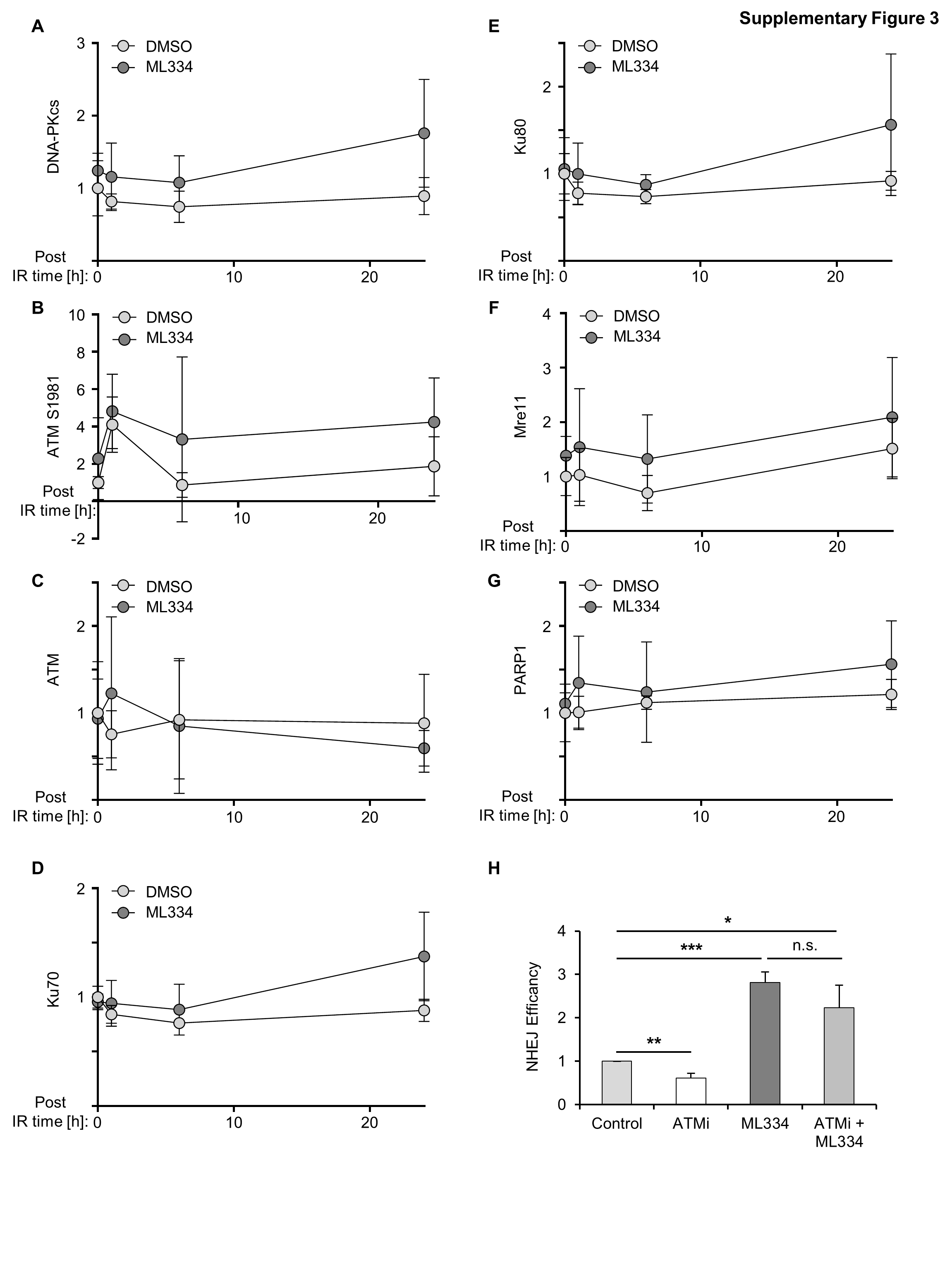

Supplement: Supplementary file 4 — Suppl. Fig. 3 [file 41419_2020_3100_MOESM4_ESM.tif]

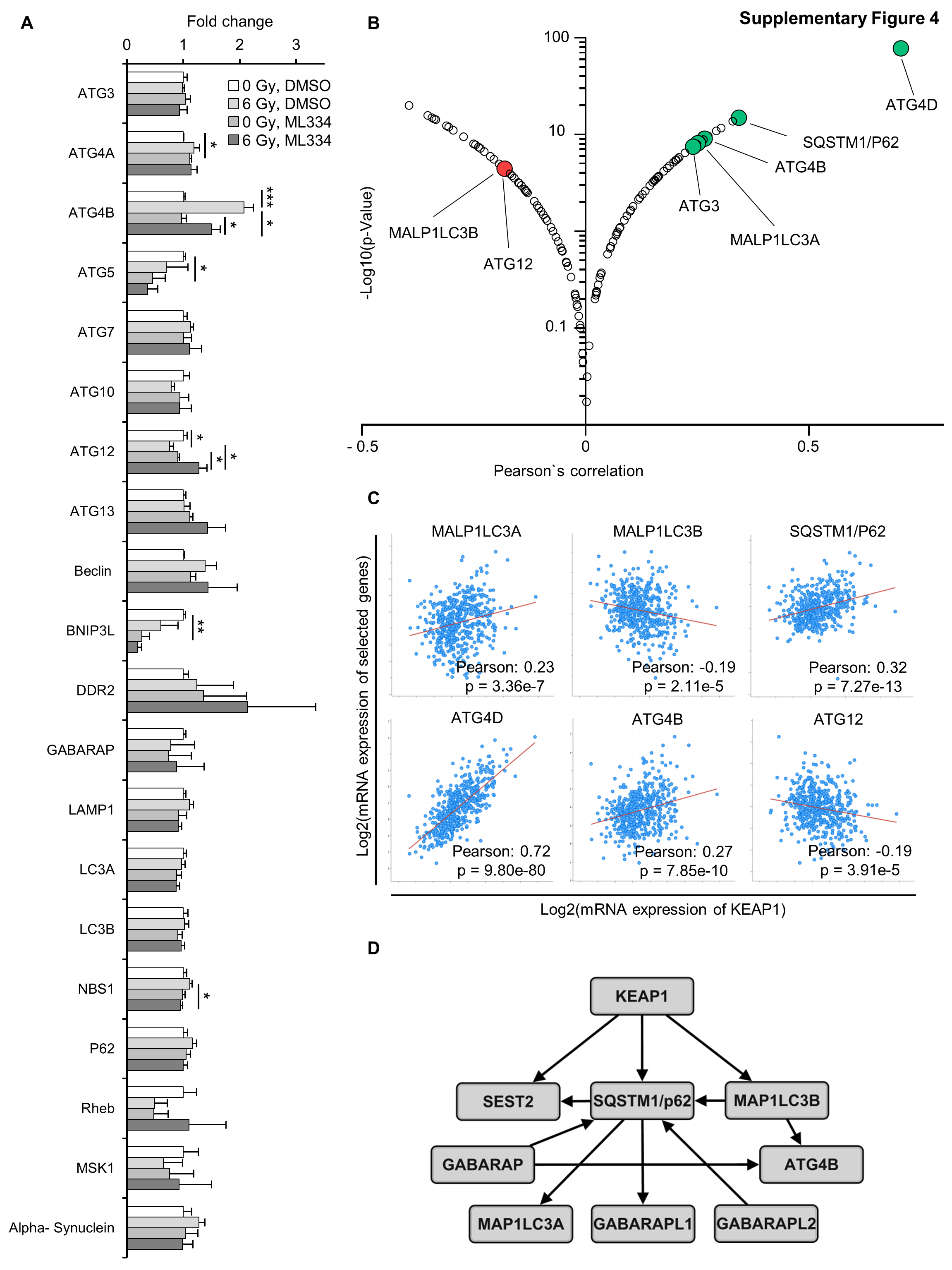

Supplement: Supplementary file 5 — Suppl. Fig. 4 [file 41419_2020_3100_MOESM5_ESM.tif]
